# Task Requirements, Workflow Processes and Technological Constraints in Virtual Multidisciplinary Tumour Board Meetings: Insights for Extended Reality-Based Solutions in Thoracic Oncology

**DOI:** 10.1093/icvts/ivag082

**Published:** 2026-03-17

**Authors:** Kathrin Adamietz, Fiona Zaruchas, Jim Krups, Jan Arensmeyer, Philipp Feodorovici, Joachim Schmidt, Matthias Weigl

**Affiliations:** Institute for Patient Safety, University Hospital, University of Bonn, Bonn 53127, Germany; Institute for Patient Safety, University Hospital, University of Bonn, Bonn 53127, Germany; Institute for Patient Safety, University Hospital, University of Bonn, Bonn 53127, Germany; Department of Thoracic Surgery, University Hospital Bonn, Lung Cancer Center Bonn/Rhein-Sieg, Bonn 53127, Germany; BOSTER—Bonn Surgical Technology Center, Bonn 53227, Germany; Department of Thoracic Surgery, University Hospital Bonn, Lung Cancer Center Bonn/Rhein-Sieg, Bonn 53127, Germany; BOSTER—Bonn Surgical Technology Center, Bonn 53227, Germany; Department of Thoracic Surgery, University Hospital Bonn, Lung Cancer Center Bonn/Rhein-Sieg, Bonn 53127, Germany; BOSTER—Bonn Surgical Technology Center, Bonn 53227, Germany; Department of Thoracic Surgery, Helios Hospital Bonn/Rhein-Sieg, Bonn 53123, Germany; Institute for Patient Safety, University Hospital, University of Bonn, Bonn 53127, Germany

**Keywords:** multidisciplinary tumour board meetings, cognitive task analysis, extended reality, XR

## Abstract

**Objectives:**

Multidisciplinary tumour boards are an essential step of high-quality, team-based decision-making in cancer care. The digitalization of distributed tumour boards enables collaboration across institutions but introduces challenges due to limited visualization and interaction. This study investigates key tasks and requirements for videoconference multidisciplinary tumour boards, examines how current technologies affect workflow and teamwork and explores requirements for future extended reality supported tumour boards.

**Methods:**

A cognitive task analysis was conducted, involving standardized tumour board observations and semi-structured clinician interviews. Key aspects such as tasks, requirements, workflow interruptions, teamwork and usability were assessed. Observational and interview data were analysed using quantitative and qualitative content analysis.

**Results:**

We observed 5 videoconference multidisciplinary tumour boards, including 146 patient cases, and interviewed 7 clinicians. We identified key steps and workflows, found frequent disruptions due to connectivity, imaging and audio issues, causing delays and hindering collaboration. Clinicians’ interviews highlighted future requirements for augmented reality-facilitated tumour boards.

**Conclusions:**

This study addresses the research gap on how tumour board technologies affect clinical workflow and teamwork. Our empirical findings corroborate that current communication tools have distinct limitations in supporting effective workflows and teamwork and highlights opportunities for future extended reality supported tumour boards.

## Graphical abstract

**Figure:**
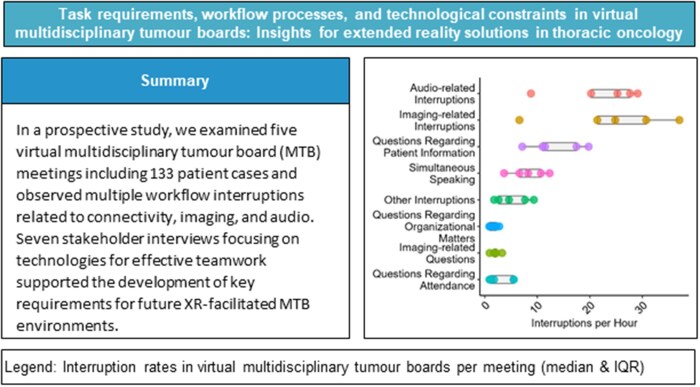

## INTRODUCTION

Cancer care requires a multidisciplinary approach to provide patients with the best possible care.^1^ Multidisciplinary tumour board (MTB) meetings are a cornerstone of cancer care and a well-established part of team-based decision-making in oncological health care.^2^ MTBs allow multidisciplinary review and discussion of patient care, fostering high-quality treatment recommendations, transparency, and knowledge exchange with well-documented benefits for decision-making and patient survival.^3–6^ The COVID-19 pandemic accelerated the adoption of videoconference MTBs (vMTBs), improving accessibility, cross-institutional collaboration, attendance, and case volume.^3^^,^^7^^,^^8^

However, vMTBs also introduced new technologies, altered work and communication flows, and imposed new challenges upon clinicians. Desktop-based tools limit visualization and interaction, which can affect teamwork and decision-making.^9^ Increased attendance does not guarantee participation, because videoconferences can hinder teamwork, communication, and attentiveness due to missing visual and tactile cues and technical issues.^10^^,^^11^ Thus, the introduction of vMTBs has brought both opportunities and challenges, highlighting the need to examine the impact of these technological changes on clinical collaboration.

Systematic investigations into the specific tasks, workflows, and requirements of vMTBs remain limited.^12^^,^^13^ There is a lack of observational studies examining how current technologies influence clinical workflows or contribute to interruptions during vMTBs.^10^ This study therefore analyses how technology influences clinical workflows, identifies flow disruptions, and explores whether existing platforms meet the demands of multidisciplinary case discussions. By combining observations and clinicians’ perspectives, our goal was to inform technological developments that enhance efficiency, collaboration, and decision-making.

Extended reality (XR) offers a potential solution to current vMTB limitations by supporting enhanced collaboration, information display, and decision-making. However, clinicians’ needs and requirements for XR-facilitated MTBs have yet to be investigated.

To address these aspects, the study employs a cognitive task analysis with the following objectives (research questions [RQ]):

- RQ1: Identify key tasks and requirements essential for effective workflow processes and teamwork in distributed vMTB meetings.
- RQ2: Assess how the technologies currently used in distributed vMTB meetings facilitate or hinder effective workflow processes and teamwork.
- RQ3: Determine key task, technology, and teamwork requirements for future XR-facilitated MTB environments.

## METHODS

### Study design

This study employed a mixed-methods design using a cognitive task analysis, integrating document review, vMTB observations, and clinician interviews. Data were collected from August to November 2024, following approval by the Ethics Committee of the Medical Faculty at Bonn University (2024–97-BO). The study was registered with the German Register for Clinical Trials (DRKS00034802).

Participants were informed about the study’s purpose and data confidentiality. They provided verbal and written consent. No personal information was collected from patients or participants in the vMTB; therefore, patient consent was not required for this study. Reporting follows the Consolidated Criteria for Reporting Qualitative Research Checklist (Materials SA).^14^

### Setting and participants

The authors investigated lung cancer vMTBs within a comprehensive cancer centre consortium (university hospital, non-academic hospitals, and outpatient providers) in North Rhine-Westphalia, Germany. At weekly meetings, they reviewed all suspected thoracic cancer cases (on average 1500 cases/year, approximately 29 patient cases per week with 31 clinicians attending).

Clinicians attend vMTB meetings remotely using an internal video-conferencing platform. Eligible participants attended at least 12 vMTBs in the past 6 months to ensure regular and experienced participants and possessed a minimum German language proficiency of B2.

### Procedure

Study information was communicated via email to vMTB attendees. Participants were recruited through email and provided with study information, voluntary participation, and data protection measures. Semi-structured interviews with senior clinicians from key specialties and roles were conducted in person or by phone and audio-recorded. The first author (KA), a female researcher with a master of science degree in global health and psychology, conducted all interviews. The vMTB sessions were observed using a semi-structured guideline by KA, with 2 trained researchers (JK, a master student in psychology, and FZ, a student in medicine). Additionally, KA observed clinicians on-site in their offices during vMTBs.

### Measures

#### Clinician interviews.

The interview guide was developed from exploratory interviews with 3 specialists and tested with 2 clinicians (Materials SB). Interviews were designed to last 45 min.

Interviews covered topics related to vMTBs: Demographics; tasks and task requirements; vMTB workflow; communication and decision-making; usability and satisfaction; and perspectives on XR-based MTBs.

#### vMTB observations and on-site observations of clinician workplaces.

The observation guide was developed from unstructured observations and the Metric for the Observation of Decision-Making II and tested on site with a specialist (Materials SC).^15^

Structured observations of vMTBs assessed the following: Meeting duration and task frequency—total meeting and task duration (in min), frequency of sub-tasks; workflow disruptions (number and frequency of disruptions caused by technical issues); participation. On-site observations focused on individual work-settings, requirements, tools, and workflow.

#### Data analyses.

All interviews were audio-recorded and transcribed. Anonymized transcripts were analysed with MAXQDA 2024 (VERBI Software, 2021). A structured, qualitative content analysis was performed using a step-wise process.^16^ First, key content categories were deductively derived and agreed on, and documented within a coding system. Second, 2 researchers (KA, FZ) independently coded all transcripts and discussed coded segments using a consensus-oriented approach.

All observations were reviewed after the meetings and discussed by 2 researchers (KA, FZ) until consensus was reached; formal inter-rater reliability was not assessed. Descriptive statistics of observational data were computed using R, version 4.5.0. Due to small meeting size and potentially skewed values, medians and interquartile ranges (IQR) were determined. For duration measures, bootstrap confidence intervals with number (*n*) of 1000 samples were used. The summarized dataset is available at https://osf.io/erjgq/overview?view_only=474680b7e60146a5b756638474a3b4d3.

## RESULTS

### Key characteristics of interviewed clinicians and observed vMTBs

Structured observations of 5 vMTBs included 146 patient cases, with a median of 34 participants per meeting (IQR: 33-36). Six on-site observations of individual clinicians participating in vMTBs were carried out; the clinicians included representatives from thoracic surgery, radiology, radiotherapy, pathology and oncology, and the meeting coordinator.

Of 12 contacted clinicians, 7 consented to interviews, representing all key specialties present in MTBs (median duration: 45.47 min, IQR = 43.99-51.95), and 6 participated in individual on-site observations. Observed participants were from 3 hospitals and 1 independent, outpatient facility, with at least 8 years of experience (median: 15 years, IQR: 11-19) and regular vMTB attendance.

Findings are presented according to the research questions, covering overall meeting requirements and workflow, facilitators and barriers, and clinicians’ perspectives on implementing XR-facilitated MTBs. Between-meeting differences are presented in Materials SF.

### RQ1: Key tasks and requirements essential for effective workflow processes and teamwork in vMTBs

During vMTBs, specialists from various clinics and disciplines participated remotely via an internal videoconferencing platform, without patients present. Attendees received patient lists in advance and typically joined from their offices via desktop workstations, which differed across institutions. A median of 14 participants (IQR: 14-15) had cameras on per meeting. Screen-sharing was limited to 1 screen at a time, including a participant list to identify attendees.

**Figure 1.**
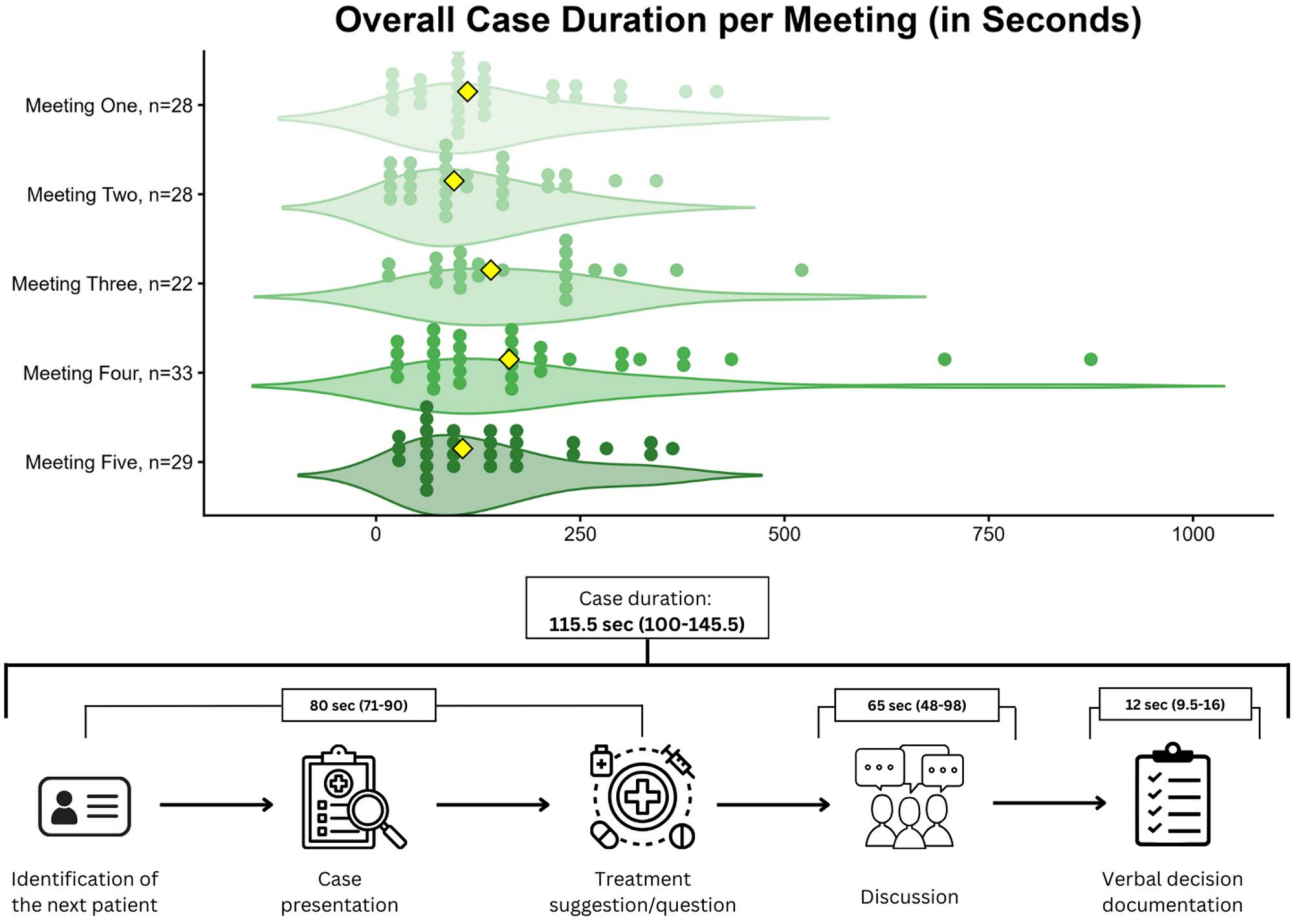
Workflow of 5 Videoconference Multidisciplinary Tumour Boards with 140 Patient Case Discussions, Including Median Durations and 95% Bootstrapped Confidence Intervals (in Brackets) in Seconds (*n* = Number of Reviewed Patient Cases)

Observed vMTBs lasted a median of 67.23 min (bootstrapped CI, 54.67-109.26) per meeting, with each case discussed for 1.93 min (CI, 1.67-2.42). The workflow comprised 5 steps, not always followed sequentially or fully completed. The typical sequence and corresponding median durations are presented in Figure 1. The frequency of observed steps of all cases, in which a case presentation took place (*n* = 133), was as follows: patient history (98.50%), radiology (82.71%), pathology (32.33%), psycho-social factors (7.58%), comorbidities (30.83%), and patient perspective (9.16%). Team discussions occurred in 56.39% of the 133 cases, typically involving 3 participants (IQR: 2-4; maximum: 8). Radiologists presented images, and documentation was provided by the coordinator with support from a documentation assistant. In 70.68% of the 133 cases, the final clinical decision for documentation was explicitly verbally stated. Based on interview data, we further characterized the typical vMTB workflow, including specific tasks, requirements, and tools for each specialty (Materials SD).

### RQ2: Facilitators of and barriers to vMTBs

We analysed clinicians’ experiences with vMTBs to identify factors that facilitated or hindered effective workflow and teamwork. These factors were categorized into 3 domains: contextual (relating to elements before and after the meeting), facilitators of intra-vMTB collaboration (occurring during the meeting itself), and technology-related facilitators or barriers.

Reported facilitators of vMTBs (Table 1) included elimination of travel requirements, improving accessibility and attendance, and enabling participation from additional hospitals and specialties. Clinicians reported improved work-life integration, particularly beneficial for part-time staff, enhanced collaboration, and overall efficiency of case discussions. Furthermore, technological facilitators such as the user-friendly videoconferencing platform and direct access to patient records and online resources were highlighted.

**Table 1.** Advantages and Technological Facilitators of Videoconference Multidisciplinary Tumour Boards

Facilitators of vMTBs	Description	No. of mentions
**Contextual facilitators of vMTBs**		
Elimination of travel requirements	Remote participation removes the need for travel, saving time and resources.	5/7
Improved accessibility and attendance	vMTBs enable collaboration across geographically distributed hospitals and clinicians and across specialties.	5/7
Better integration into the workflow	Online meetings require less time overall and are less disruptive to clinical workflows. Time before and after meetings can be used for other tasks.	2/7
Organizational efficiency	Centralized virtual platform fosters coordination. Preparation time is reduced because imaging and documents are shared digitally, eliminating the need for physical media.	2/7
Better work–life integration	Participation is possible from various work settings (eg, home offices), improving compatibility with personal schedules.	1/7
Minimal technical requirements	Requires only standard equipment (eg, a laptop and internet), which most participants already have.	1/7
**Facilitators of intra-vMTB collaboration**		
Enhanced collaboration	Improved collaboration across institutions and specialties, enhancing discussion quality and decision-making.	4/7
High efficiency	Despite the involvement of numerous participants, many cases can be discussed efficiently within a short time frame, with discussions remaining productive and focused. Simultaneous presentations by the referring clinician and image demonstrations by the radiologist reduce meeting duration.	3/7
Discussion of more patients	Because individuals from more hospitals are attending, more patients can be discussed with input from a broader professional base.	2/7
Flattened hierarchy	Virtual formats promote balanced discussions and encourage broader participation. Interactions tend to be more respectful, even in cases of disagreement.	1/7
**Technological facilitators of vMTB**		
Screen sharing	Enables all participants to share information or images.	3/7
User-friendly platform	Reliable and intuitive platform interface facilitates smooth communication.	3/7
Access to patient data	Allows the retrieval of patient data or studies in real time during meetings.	3/7
Effective image visualization	Supports accurate case analysis.	1/7
Attendance list	Allows everyone to see which clinicians are participating in the meeting.	1/7
Camera use	Maintaining video presence improves communication and teamwork.	1/7

Abbreviation: vMTB, videoconference multidisciplinary tumour boards.

Beyond the meeting format, several elements were identified as critical for meeting quality and efficiency. These included standardized registration of cases with required information to support structured preparation, as well as the presence of a dedicated MTB coordinator, thorough preparation by the presenter and availability of complete patient information.

Barriers to vMTBs (Table 2) included reduced personal and informal interactions. Challenges related to limited feedback and non-verbal communication, along with lower engagement, often were exacerbated by participants keeping cameras off. Technical barriers included slow or unstable connections, which led to poor audio or image quality and were frequently reported. Furthermore, some also criticized the small image size. Clinicians emphasized that, regardless of the meeting format, missing or incomplete patient data were seen as a major barrier to decision-making. Clinicians’ perspectives differed: Some felt that the virtual format increased participation, whereas others experienced it as a barrier. The need to unmute oneself before speaking and the more passive nature of virtual attendance were seen by some participants as factors that discouraged active engagement.

**Table 2.** Disadvantages and Technological Barriers of Videoconference Multidisciplinary Tumour Boards

Barriers to vMTBs	Description	No. of mentions
**Contextual barriers to vMTBs**		
Reduced informal and social interaction	Limits informal, ad hoc professional and social exchanges that may typically occur before and after in-person MTB meetings.	4/7
Variable work environments	Differences in technical set-ups (eg, internet speed, monitor size, audio-visual quality) may negatively impact meeting quality.	1/7
**Barriers to intra-vMTB collaboration**		
Reduced non-verbal feedback	Facial expressions, gestures and immediate reactions are reduced or absent, hindering dynamic and nuanced communication.	3/7
Less personal engagement	Reduced camera use by participants, further reducing interaction and making it unclear who is actively participating.	3/7
Higher participation barrier	Some participants may feel less confident or hesitate to speak up in a virtual setting, particularly when cameras are off or the group is large.	1/7
**Technological barriers in vMTBs**		
Connectivity and technical disruptions	Poor internet connections, updates, software crashes and server issues can interrupt access to essential data or delay meetings.	7/7
Poor image quality/resolution	Small screens, inconsistent resolution due to internet issues and non-optimized sharing impair interpretation of radiological images.	6/7
Audio quality problems	Poor microphone etiquette (eg, failing to mute) causes background noise, echo and disrupted meeting flow and concentration. Failing to unmute can lead to omission of key information.	5/7
Disrupted speaking flow	Lack of visual cues and turn-taking structure can result in overlapping speech and missed contributions.	3/7
Protocol not shared in real time	When protocols are not shared, reliance on verbal communication increases the risk of missing critical information, especially during distractions.	3/7
Delayed transitions during screen sharing	Switching between patients leads to longer image loading times, and delayed switching between presenters can disrupt flow and efficiency.	1/7

Abbreviation: MTB, multidisciplinary tumour board; vMTB, videoconference multidisciplinary tumour board.

### Observed interruptions of vMTBs

**Figure 2.**
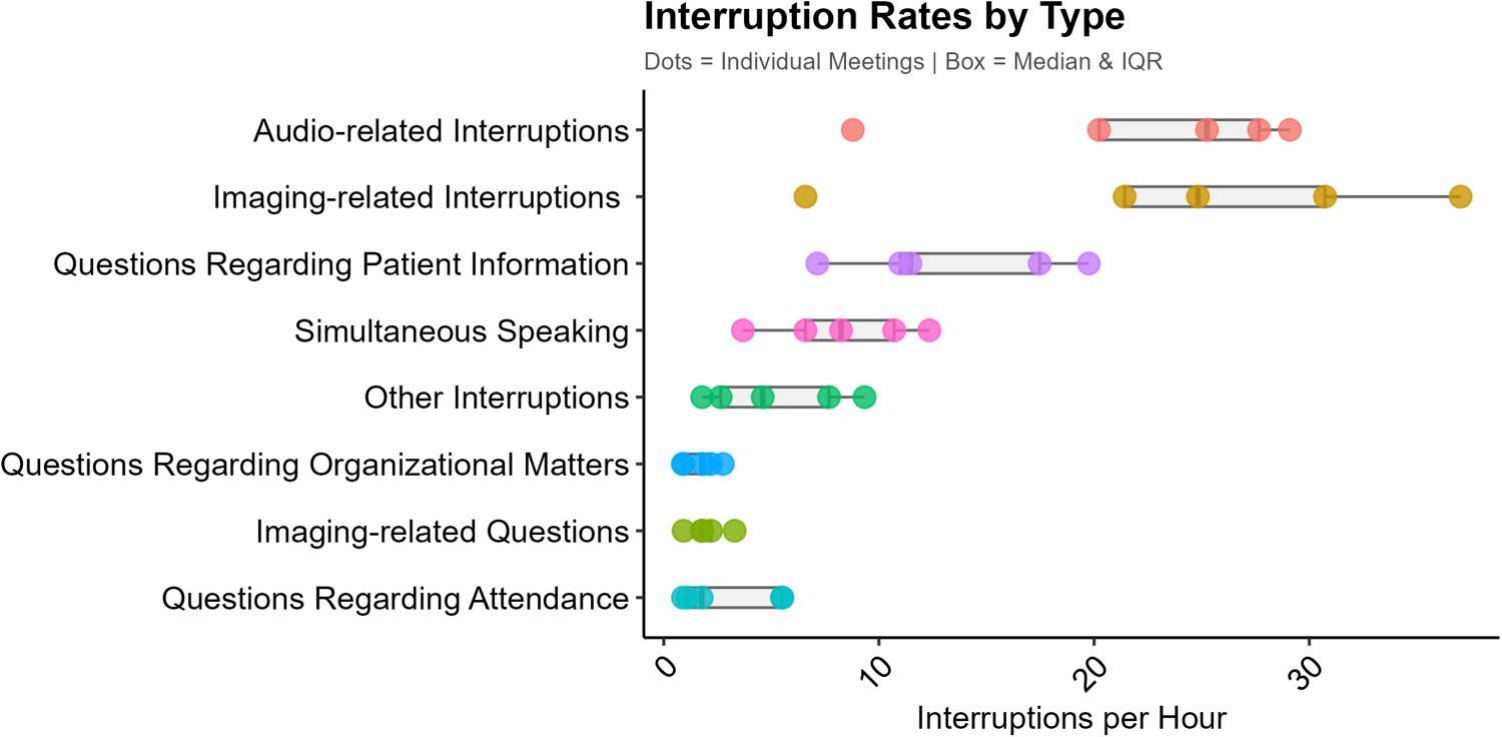
Rates of Observed Workflow Interruptions and Reasons for Questions During Videoconference-Based Multidisciplinary Tumour Board Meetings (*n* = 5)

These barriers were also evident during vMTBs (Figure 2), with technical interruptions being present a median of 78.16 times per hour (bootstrapped CI, 73.18-97.89). Of 6.07 hours of observed vMTBs, frequent workflow interruptions were caused by technical issues, including image-related problems (*n* = 133, *n*/hour = 24.83, CI, 21.42-30.73) and audio disruptions (*n* = 126, *n*/hour = 25.24, CI, 20.23-27.66) or through simultaneous speaking (*n* = 51, *n*/hour = 8.24, CI, 6.59-10.71). Additional disruptions arose from clarification questions or information seeking, such as inquiries about patient information (*n* = 30, *n*/hour = 11.47, CI, 10.98-17.47) or clinician attendance (*n* = 16, *n*/hour = 1.76, CI, 1.10-5.49). Notably, there was considerable variability in interruption rates across meetings (Materials SF).

### RQ3: Clinicians’ perspectives on implementation of XR-facilitated MTBs

Of the clinicians, 3 had used XR in their professional work, 3 had some private experience and 3 had no prior experience. Concerning our third research question, we classified interviewees’ statements into the following major categories:

### Technical improvements to current vMTB

Though clinicians were generally satisfied with the current vMTB format, they recommended improvements. A virtual “raise hand” function was suggested to improve turn-taking and reduce disrupted speaking flow. Additionally, they recommended integrating a chat function to enable discrete, direct communication between participants, reducing the barriers for speaking up and facilitating professional exchange. Most frequently mentioned was the option to share the protocol, allowing participants to access key clinical information. This was conceived as particularly useful when details were missed due to distractions or technical issues during a case presentation. Current platforms’ single-screen sharing limited these capabilities.

### Expected benefits, concerns, and requirements of XR-facilitated MTBs

The majority of clinicians interviewed deemed that XR technology could offer a promising alternative to overcome current barriers. XR technology may enhance imaging and information sharing beyond the constraints of desktop-based platforms, improve communication and create a more personal and interactive meeting environment (see Table 3 and Materials SE for descriptions).

**Table 3.** Clinicians’ Expected Benefits of Participating on Extended Reality-Facilitated Multidisciplinary Tumour Boards

Expected benefits of XR-facilitated MTBs	No. of mentions
Enhanced image visualization	3/7
Better decision-making and improved patient safety	3/7
Advanced information sharing	2/7
Enhanced communication and personal experience	2/7
Shorter and more effective case discussions	2/7
Promotion of the educational character of meeting	2/7
Use of technological advancements in XR	1/7

Abbreviation: XR, extended reality.

However, clinicians also expressed concerns regarding the implementation of XR technologies (Table 4; see Materials SE for descriptions). Six key requirements for safe and effective XR development and implementation were identified: (1) Data protection and security (mentioned by 5/7 interviewees); (2) training (5/7); (3) financial resources (4/7); (4) added value (3/7); (5) technical and personnel support (3/7); and (6) legal feasibility (2/7) (see Materials SE for details).

**Table 4.** Clinicians’ Concerns Regarding XR-Facilitated MTBs

Implementation concerns for XR-facilitated MTBs	No. of mentions
Feasibility and implementation effort	5/7
Decreased workflow efficiency	4/7
Data privacy and security risks	4/7
Limited impact on decision-making	3/7
Limited staff acceptance	3/7
Distraction and potential hindrance	2/7
Reduced precision in visualization	1/7
Potential patient safety risks	2/7

Abbreviations: MTB, Multidisciplinary Tumour Board; XR, extended reality.

## DISCUSSION

Efficient teamwork and communication within MTBs are key components of high-quality cancer care. Successful teamwork is particularly critical in vMTBs, imposing further constraints due to novel technologies and altered modes of communication and interaction. This study presents a mixed-methods evaluation of vMTBs in thoracic oncology, examining technological, teamwork and workflow factors. By integrating clinician interviews and observations, we identify key tasks, highlight facilitators and barriers in current practice and explore the potential of XR in addressing existing constraints.

Patient discussions lasting a median of 1.9 min may appear brief: Mandatory review of all thoracic cancer cases necessitates shorter discussions for routine cases. Comparable discussion lengths have also been reported in previous studies.^17^ To maintain efficiency, clinicians typically present only information relevant to the decision at hand, for example, patients’ special circumstances or treatment preferences that diverge from the clinician’s initial recommendation. This selective reporting may explain variations in step-completion rates; however, this also introduces subjectivity. The high passive participation may reflect both multi-institutional attendance and authority or experience gradients in decision-making.

Clinicians reported overall satisfaction with the vMTBs, describing them as a viable evolution of in-person MTBs. Key facilitators included improved accessibility, elimination of travel, opportunities to discuss more cases and enhanced work-life balance, consistent with results from previous studies.^3^^,^^7^^,^^13^ Rehman et al.^13^ reported improved attendance and reduced preparation time, whereas Davis et al.^3^ found a 46% increase in attendance and a 20% rise in cases discussed compared with traditional MTBs.

vMTBs impose constraints on collaboration and efficiency, with frequent technology-related interruptions, including unstable internet and audio-visual issues. Other interruptions were less frequent and did not necessarily disrupt discussion flow, suggesting that some interruptions were managed without substantially impairing conversational continuity. Whereas Soukup et al.^18^ attributed decision delays to hybrid disconnects, our observations indicated that software issues were the main cause. Groothuizen et al.^19^ observed fewer interruptions, likely due to their narrower definition of “disruption.” Our broader approach captured a more comprehensive picture of technological and flow constraints. Differences in connectivity and equipment across participating institutions further influenced clinicians’ ability to engage effectively. Even after years of implementation, such interruptions persist, hindering workflow, delaying decisions and prolonging meetings. Interviews confirmed that reduced informal and non-verbal communication limits interaction quality, echoing Rehman et al.^13^ Hybrid formats require further evaluation, as they preserve in-person interaction for clinicians who are physically present, yet they also introduce drawbacks that can reduce discussion quality and weaken team cohesion.^12^^,^^13^

### Implications for clinical practice

Because distributed vMTBs offer several advantages over in-person meetings, including improved accessibility and stronger connections between regional multi-institutional teams, thereby supporting more equitable cancer care, the continued development of optimized vMTB environments is warranted, whether fully virtual, hybrid or XR-based.^13^ Our findings underscore the need to critically assess current vMTB teamwork practices and explore strategies to align technologies with the demanding requirements of multidisciplinary case discussions. We recommend (1) multiscreen sharing including the protocol to facilitate the simultaneous review of imaging and patient information; (2) structured communication tools (eg, “raise hand” functions); (3) enhanced visual presentation, because radiologists should prioritize displaying most relevant images (typically 1 or 2) and allow key participants, such as surgeons, to access imaging directly; (4) good meeting etiquette (i.e., muting, turn-taking) and (5) improved bandwidth to minimize interruptions.

Although chat features may support interaction, they risk fragmenting discussion and reducing transparency. Thus, new tools must align with clinical workflows and collaborative goals. This is particularly relevant when considering more advanced tools such as XR, which clinicians viewed as promising means of overcoming current limitations in data visualization, interaction and engagement of desktop-based systems. Studies such as that by Almashmoum et al.^20^ showed that 3-dimensional views during XR-MTBs improved efficiency and created in-person engagement, though connectivity issues remain a challenge.

Clinicians raised concerns regarding XR adoption, including legal, data protection, compliance, usability and cost concerns. The implementation of XR remains exploratory and requires further validation. Future XR environments should ensure requirements of robust data protection and security, legal frameworks, adequate resources, IT support and clinical training. Development should follow participatory design principles, ensuring that XR enhances rather than replicates current workflows.

Amid rising cancer burdens, staffing shortages and financial pressures, identifying barriers to and enablers of vMTB performance supports quality improvement in oncology.^21^ The strength of this study lies in combining observations and interviews to examine real-time workflow dynamics and interruptions. It is the first observational study to specifically investigate workflow interruptions in vMTBs, with a particular focus on the role of technology and communication. Including both distributed clinicians in and outside hospitals, it allows enhanced generalizability across care settings. This study combines insights from current vMTBs with considerations for the development of XR-supported MTBs.

### Limitations

Nevertheless, several limitations must be acknowledged. To minimize the Hawthorne effect, vMTBs were observed over an extended period. Observations and interviews focused on clinicians involved in vMTBs, ensuring relevance and depth of insight. Despite participants’ extensive vMTB experience, the limited interview sample and single regional thoracic oncology setting may have constrained thematic breadth and transferability.

Selection bias may have led to participation of individuals more concerned about current limitations or more enthusiastic about XR. Live observations without recordings may have introduced minor inaccuracies or data gaps, and the complementary nature of observations might have introduced observer bias. Furthermore, we did not include the complexity of patient cases in our observations nor are we able to judge the quality of decisions made during the vMTBs. We did not include clinical outcomes, such as the adherence to clinical guidelines or mortality outcomes.

Future studies should record MTBs and evaluate inter-rater reliability for coding observational data. Recording would also enable interruptions to be classified by severity and duration and allow evaluation of whether interruptions lead to deferred decisions or prolonged discussions. Incorporating case complexity as a contextual variable would further strengthen analyses. In addition, larger samples and the reporting inter-coder agreement metrics are needed to improve the robustness and reliability of qualitative analyses.

## CONCLUSION

This study was designed to investigate facilitators and barriers to teamwork and workflow of current vMTBs, offering valuable insights for improvement and laying the foundation for future MTB tools in oncology. Although videoconferencing enhances accessibility, it also introduces novel barriers to interaction, workflow and collaborative decision-making. Extended reality offers a promising technological direction, but its development must be carefully guided by user-centred design, clinical task alignment and demonstrable value. Optimizing future vMTBs requires technological innovation aligned with clinical processes, teamwork dynamics and user-centred design.

## Supplementary Material

ivag082_Supplementary_Data

## Data Availability

The quantitative data and analytic code underlying this article is available in the Open Science Foundation in aggregated, meeting-level form, including interruption counts and rates per meeting and all summary statistics per meeting (https://osf.io/erjgq/overview?view_only=474680b7e60146a5b756638474a3b4d3). Only process data is available—no personal participant data. The qualitative data cannot be shared publicly because of the need to protect the privacy of the individuals who participated in the study.
