# Supplementary material for: Task Requirements, Workflow Processes and Technological Constraints in Virtual Multidisciplinary Tumour Board Meetings: Insights for Extended Reality-Based Solutions in Thoracic Oncology
Source: Interdiscip Cardiovasc Thorac Surg. 2026 Mar 17;41(4):ivag082. doi: 10.1093/icvts/ivag082 (PMC13094740; doi:10.1093/icvts/ivag082)
Supplement: ivag082_Supplementary_Data [file ivag082_supplementary_data.doc]

**
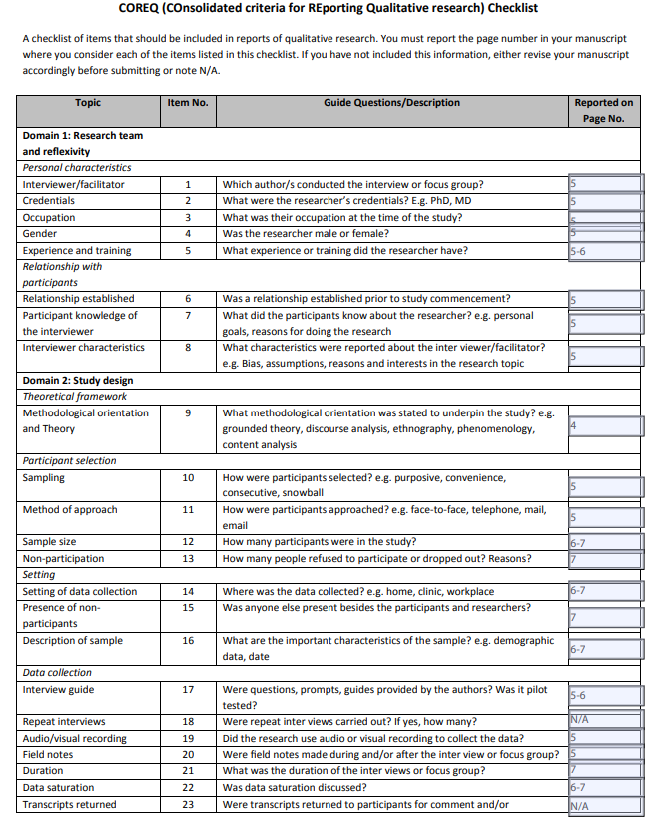
Supplementary Materials A**


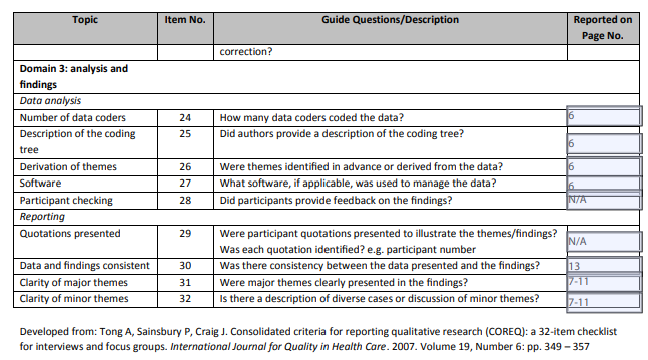


**Supplementary Materials B**

**Interview Guide**

Task requirements and usability of a virtual thoracic tumor board

Thank you for agreeing to be interviewed for this research project. We are interested in identifying the work process, associated task requirements, and usability of a tumor board. The interview should take about 45 minutes.

Is it alright if I record the interview?

Do you have any questions before we begin?

Demographic questions

What is the name of your position?

How long have you been working in this department?

How many years of professional experience do you have in your field?

How long have you been participating in virtual tumor boards?

How many tumor boards have you participated in approx. over the last 6 months?

On a scale of one to ten, how would you assess your level of digital proficiency?

Task requirements of a tumor board

1. What preparations for a tumor board need to be made and by whom? *Review of clinical tests & notes, software*
2. What tasks/work steps are relevant during a tumor board meeting and who is responsible for them? *Different roles*

Workflow

1. How does a regular tumor board meeting proceed, in what order are the steps carried out, and when is a step considered complete?
2. Which tools or software programs are required for a tumor board (e.g. JOIN, CT images, electronic patient record, protocol) and which participant is responsible for each of them?
3. How do technology and software programs support or disrupt the tumor board?
4. Are there any reasons why you need to divert your attention away from the discussion during the tumor board?

Communication and decision-making

You are often in a team of around 30 people, but considerably fewer participate.

1. Who is actively involved in the discussion? Which members actively participate in the decision-making process? (How are responsibilities allocated or communicated?)

I noticed that not for all patients the same amount of information is shared, such as medical history, psychological/social factors, comorbidities, patient wishes and discussion.

1. Why is that, and how is the relevant information shared with you? Medical record/Protocol access (parallel data projection)?
2. How do you make sure all the important information is shared?
3. What are the most common reasons for questions or interruptions?
4. What decisions need to be made?
5. If there are different opinions, how do you make a joint decision?

- For young doctors: Would you get involved in the conversation if you still had concerns?

1. What do you personally gain from the tumor board?

Usability and satisfaction

1. How satisfied are you with the overall tumor board process? What works well and what could be improved?
2. Has anything changed since tumor boards started being held virtually? Reference to collaboration & communication
3. What advantages and disadvantages do you see in tumor boards being held in person/virtually?

- Attendance, participation, communication, image visualisation, access to information, efficiency, protocol

1. How satisfied are you with the technical functions of the software used for tumor boards and its usability? (Can you see the images clearly?) (How do you use the software (image presentation, zoom, scroll, arrows, mouse, image selection))
2. In your opinion, which aspects of the software or presentation could be improved?
3. What other aspects could a tumor board benefit from?

Attitude towards augmented reality

Now I would like to ask you a few questions relating to the further development of the existing tumor board in extended reality, i.e. virtual reality and augmented reality.

1. Have you ever had contact with any form of extended reality (XR), i.e. virtual reality, augmented reality or mixed reality?

Explanation, if necessary:

I would like to present a possible further development of the tumor board using extended reality, i.e. virtual reality and augmented reality. This would involve switching from a desktop-based display to head-mounted displays, i.e. displays attached to the head. These technologies offer the possibility of immersing yourself in a virtual environment with your colleagues. It allows layered CT images to be displayed in three dimensions and navigated and edited interactively. Think about the current tumor board being further developed for the future.

1. What do you think about the integration of extended reality technology into tumor boards?
2. What features would you like to see in an immersive tumor board?
3. How do you think the integration of XR into tumor boards would affect your working methods and results?
4. Would the visualisation using XR better meet the requirements for decision-making?
5. How do you estimate the amount of work involved in integrating XR into tumor boards?
6. How do you think your colleagues would assess the integration of XR into tumor boards?
7. What resources would simplify the implementation of XR in your opinion?
8. What opportunities and risks do you see?
9. What impact on patient safety do you expect?

Do you have any questions for us?

Thank you for participating!

**Supplementary Materials C**

**Observation guide for multidisciplinary thoracic tumor boards**

Patient: Date:

| Tasks | Yes/ No | Tools/Technology (public) | Interruptions | Active participants |
| --- | --- | --- | --- | --- |
| Log in to the meeting |  |  |  |  |
| Identification of the next patient |  |  |  |  |
| **Case demonstration by clinician** |  |  |  |  |
| Treatment history |  |  |  |  |
| Radiology (X-Ray) |  |  |  |  |
| Pathology |  |  |  |  |
| Psychological/social aspects |  |  |  |  |
| Comorbidities |  |  |  |  |
| Patient perspective/-wishes |  |  |  |  |
| **Formulation staging** |  |  |  |  |
| **Treatment suggestion/ question** |  |  |  |  |
| **Discussion** |  |  |  |  |
| **Documentation of decision** |  |  |  |  |

Comments:

**Observation guide for multidisciplinary thoracic tumor boards**

Patient: Date:

Participants (with camera) at the beginning: after 15 min:

| Tasks | Reasons for questions/ interruptions | | | | Tools/Technology | |
| --- | --- | --- | --- | --- | --- | --- |
|  | Public | | Individual | | Public | Individual |
| Log in to the meeting |  | Time |  | Time |  |  |
| Identification of the next patient |  |  |  |  |  |  |
| Case demonstration  +  Treatment suggestion/ question |  |  |  |  |  |  |
| Discussion |  |  |  |  |  |  |
| Documentation of decision |  |  |  |  |  |  |

Comments:

**Observation guide for multidisciplinary thoracic tumor boards**

Date:

| **Patient** |  |  |  |
| --- | --- | --- | --- |
| **Time** |  |  |  |
| **Task** |  |  |  |
| Identification of the next patient  &  Case demonstration |  |  |  |
| Treatment suggestion/ question |  |  |  |
| Discussion |  |  |  |
| Documentation of decision  &  End |  |  |  |

Comments:

**Supplementary Materials D**

|  |  | **Radiology** | **Pathology** | **Pneumology** | **Oncology** | **Thoracic surgery** | **Radiation Therapy** | **Coordinator Lung Cancer Centre** |
| --- | --- | --- | --- | --- | --- | --- | --- | --- |
| **Patient registration** | **Tasks** | Patients with suspected or confirmed lung carcinoma must be registered at the tumorboards. Registration is only possible once all relevant clinical information is available.  Patients are registered twice (per E-Mail and in the documentationsystem Celsius 37) The final patient list is distributed to all participants one day prior to the meeting | | | | | | |
|  | **Tools** | ● Documentationsystem Celsius 37 | | | | | | |
| **Case Preparation** | **Tasks** | Preparation (In-house Cases Only) ● Check whether imaging was performed in-house or externally ● Review CT or MRI scans and classify findings according to clinical TNM staging ● If clinical records are available, compare them with imaging findings to assess consistency, re-measure if necessary to ensure alignment with clinical classification ● Prepare image demonstration for the MTB | ● Upon receiving the patient list, a technical assistant verifies whether sample material has been received and records the sample number, which is then forwarded to the pathologist  ● The pathologist reviews the clinical records for completeness and identifies any open questions or points for discussion ● If records are missing, they note expected delivery times or consult colleagues to check whether a report has already been completed and which clinician is responsible for the case | Often leading case presentations Presentations are prepared and led by the attending physician; if unavailable, another clinician prepares the case and consults colleagues regarding open questions. Preparation involves pre-filtering, summarizing, and integrating all relevant information to assess the overall clinical picture: ● Determine familiarity with the patient ● Review and interpret the patient’s medical history and clinical records, including pre-existing conditions ● Assess tumor stage and develop the preliminary tumor classification by reviewing imaging and clinical records, potentially re-measuring, and checking for distant metastases ● Confirm tumor type based on pathology ● Ensure internal consistency and coherence across all findings ● For therapy recommendations, consider previous illnesses, lung function, operability, and ECOG performance status ● Enter all relevant information into the registration system | | | ● For patients they are familiar with, clinical records and discharge summaries are reviewed to recall prior history and treatments, including any past radiotherapy—serving as a basis for future radiotherapy recommendations. | ● Tumor documentaries: Distribute the finalized patient list to all tumor board participants via email. |
|  |  |  |  |  | | ● Postoperative Patient Assessment: Assess tumor stage and evaluate pathological findings |  |  |
|  | **Tools** | ● CT, PET-CT, and/or Brain MRI● Nexus-RIS (Radiology Information System)  ● PACS (Picture Archiving and Communication System by Dedalus)  ● Segmentation tool | ● Patho-Pro: database that contains pathology reports  ● Patient list | ● Medical records (Only clinic-internal records can be accessed)● Patient list | | | | |
| **Overall Case Presentation** | **Tasks** | ● Patient is presented by the attending physician himself or a colleague: Patients are organized by clinic, then by pre- and post-surgery status, and finally in alphabetical order. ● Verbal presentation of relevant information including medical history, tumor location, size, type, formula, pulmonary function, additional diagnostic results, pathological results, comorbidities, performance status, lifestyle habits, living circumstances, patient wishes ● At the same time, the radiologist demonstrates relevant images to support the case presentation and discussion ● Most clinicians who have seen the patient open the electronic record to review relevant information and support the case discussion, such as diagnostic findings and is especially helpful when aspects of the presentation are unclear or require clarification | | | | |  | ● Opens the meeting and welcomes all participants ● Supervises documentation during the entire MTB, with active writing done by an assistant ● Documentation is done using C37 and can only be done at one location ● Monitors time and ensures efficiency; speeds up the meeting when appropriate |
|  | **Tools** | ● Conducted online through a secure, in-house platform ● Participants can join via computers, laptops, or smartphones, independent of their physical location ● Devices require a stable and fast internet connection, speakers, microphone, and a camera/webcam ● Most clinicians have a printed version of the patient list and a stable workstation ● Access to medical records is restricted to clinic-internal data only, ensuring confidentiality ● Many have one or multiple monitors to view medical records on one screen and the conference on the other to support the case discussion | | | | | | |
| **Case Presentation and Treatment Suggestion** | **Tasks** | ● Sharing of radiological images (CT/PET/MRI) in axial plane orientation for in-house patients  ● Ensure that presented information is supported by radiological imaging; make corrections to the case presentation if necessary  ● Each clinic provides its own radiologist, who shares their screen and presents the relevant images during the tumor board meeting | ● Double-check of identifying information (birth date, Name)  ● Check for duplicate entries of the patient in the system and review for any irregularities or inconsistencies  ● Provide relevant pathological information for the case | ● Often conduct case presentations  ● Otherwise actively listen to case presentation and consider if the presentation is clear or if other approaches would be beneficial too  ● Suggestion of possible treatment options | | | ● Active listening to presenting clinician | ● Identification of the next patient and passing the word to treating clinician  ● Ensure that all relevant information is being presented |
|  | **Tools** | ● Radiological images: RIS and PAX ● Medical records, radiological findings ● Patient list | ● Patho Pro ● Medical records with additional notes ● Patient list | ● Medical records with additional notes ● Patient list | | | | ● Documentation: Celsius 37  ● Patient list |
| **Discussion** | **Tasks** | ● Discussion of CT/PET scans, if necessary ● Presentation of certain images on request | ● Contribution of relevant pathological information | ● Contribution of pneumological expertise ● Corrections regarding treatment suggestion if suggested treatment conflicts with pathologicial findings | ● Provide treatment suggestions from an oncological perspective ● Evaluate systemic therapy options and their potential benefits and risks. ● Contribute relevant oncological knowledge as needed to support the discussion | ● Discussion of suggested treatment concept ● Contribution of relevant knowledge concerning possible surgeries | ● Contribution of relevant knowledge regarding radiotherapeutic treatment options ● Veto if suggested treatment does not seem plausible form a radiotherapeutic point of view | ● Ensure that a consensus regarding therapeutic and diagnostic concept is achieved ● Documentation of process of decision-making ● Documentation of diverging opinions regarding treatment options -> patient involvement in treatment decision ● Ensure that all relevant aspects of the discussion are documented |
| **Documentation** | **Tasks** |  | | | | | | ●Documentation is done by the tumor board assistant, based on the chair’s dictation  ● The chair specifies what should be included in the tumor board protocol and checks for completeness at the end  ● The protocol must include the tumor classification and the agreed-upon treatment or diagnostic plan  ● The final decision is read aloud to ensure transparency |
| **Follow-up** | **Tasks** | ● After the meeting, the finalized protocol is distributed to all participants ● Tumor board protocols can be added to the patient's medical record ● If discrepancies arise when the patient presents for treatment, the case is re-discussed in the tumor board | | | | | | |
|  |  |  |  |  |  |  |  |  |

**Supplementary Materials E**

**Table 1.** Clinicians´ expected benefits of XR-facilitated MTBs including descriptions.

| **Expected benefits of XR-facilitated MTBs** | **Description** | **# of mentions** |
| --- | --- | --- |
| Enhanced image visualization | Detailed 3D display of tumours, anatomical extensions, and vasculature for improved spatial orientation. | 3/7 |
| Better decision making and improved patient safety | Clearer decisions supported by improved information quality, leading to enhanced patient safety and overall diagnostic and therapeutic outcomes. | 3/7 |
| Advanced information sharing | XR enables simultaneous viewing of multiple virtual screens—combining imaging, bronchoscopy, reports, and patient data. | 2/7 |
| Enhanced communication and personal experience | Enables natural, interactive communication with nonverbal cues and increased feedback, fostering engagement and focus, closely resembling in-person meetings. Opportunities for enhanced comfort compared to traditional PC setups. | 2/7 |
| Shorter and more effective case discussions | Making unclear or ambiguous points more explicit to reduce misunderstandings and facilitate the discussion, resulting in more focused and efficient MTBs. | 2/7 |
| Promotion of the educational character of meeting | Improved spatial visualization of anatomical structures and direct access to recent studies. | 2/7 |
| Use of technological advancements in XR | Leveraging emerging technologies, including XR and AI, to enable accurate, structured, and intelligent presentation of clinical data. | 1/7 |

**Table 2.** Clinicians´ concerns regarding XR-facilitated MTBs including descriptions.

| **Implementation Concerns for XR-Facilitated MTBs** | **Description** | **# of mentions** |
| --- | --- | --- |
| Feasibility and implementation effort | High implementation effort required, including legal compliance, hardware/software compatibility, training and time investment. | 5/7 |
| Decreased Workflow Efficiency | MTBs in XR may slow down processes, increase cognitive load, and require more time compared to current methods. | 4/7 |
| Data Privacy and Security Risks | Potential exposure of sensitive patient data; ensuring compliance with data protection regulations is essential. | 4/7 |
| Limited Impact on Decision-Making | Enhanced visualization may not improve decision making or MTB outcomes, current imaging may already be sufficient. | 3/7 |
| Limited Staff Acceptance | XR technologies may face resistance from colleagues. | 3/7 |
| Distraction and Potential Hindrance | XR elements could be distracting or introduce technical issues that disrupt meetings or decision-making. | 2/7 |
| Reduced Precision in Visualization | 3D or XR images may compromise detail and clarity, making fine anatomical structures harder to interpret. | 1/7 |
| Potential Patient Safety Risks | Misinterpretation, visual distortion, or overreliance on XR representations could lead to inaccurate clinical judgments and negatively impact patient safety. | 2/7 |

**Table 3.** Clinicians´ requirements for XR-facilitated MTBs.

| **Implementation Concerns for XR-Facilitated MTBs** | **Description** | **# of mentions** |
| --- | --- | --- |
| Data protection and security | Robust measures must be established to ensure compliance with data protection regulations and to safeguard sensitive information. | 5/7 |
| Training | Clinicians shall receive training to confidently use and navigate the XR environment. | 5/7 |
| Financial resources | Significant investment will be required to equip participants with XR-compatible hardware. | 4/7 |
| Added value | XR must provide a clear benefit, merely replicating the existing vMTB format in XR would not justify the substantial implementation effort and associated costs | 3/7 |
| Technical and personnel support | Dedicated support staff should be available to ensure smooth operation, possibly shared across tumour boards | 3/7 |
| Legal feasibility | A clear legal framework must be in place, ideally supported by financial incentives to adopt XR . | 2/7 |

**Supplementary Materials F**

**Table 6. Variables overall and per meeting.**

| **Variables** | **Overall Meetings** | **Meeting 1** | **Meeting 2** | **Meeting 3** | **Meeting 4** | **Meeting 5** |
| --- | --- | --- | --- | --- | --- | --- |
| Clinicians per meeting | 34 | 36 | 34 | 30 | 33 | 41 |
| Patient cases per meeting | 29 | 31 | 29 | 22 | 35 | 29 |
| Meeting duration *(minutes)* | 67:38 | 68:03 | 54:67 | 65:25 | 109:26 | 67:23 |
| Patient case duration *(median in seconds,*  *Bootstrap CI 95%)* | 115.5s (100-145.5) | 112s (92-142) | 95.5s (74-149) | 140.5s (103-229) | 163s (88-194) | 106s (73-160) |
| Case presentation  *N: patient cases with case presentation*  *Median duration, Bootstrap CI 95%*  % of information presented  patient history   radiology   pathology  psycho-social factors  comorbidities  patient perspective | N=133  77s (64-85)  98.50%  82.71% 32.33% 7.58% 30.83% 9.16% | N=28  91s (58-103)  100% 92.59% 40.74% 3.70% 29.63% 11.11% | N=26  68s (52-96)  100% 73.08% 11.54% 15.39% 38.46% 0.00% | N=21  96.5s (79.5-116)  100% 80.95% 38.10% 4.76% 28.57% 19.05% | N=32  71s (61.-83)  93.75% 75.00% 43.75% 9.38% 31.25% 15.63% | N=27  76s (60-92)  100% 92.59% 25.93% 3.94% 25.93% 0.00% |
| Team case discussion  *N: patient cases with discussion*  *Median duration in seconds, Bootstrap CI 95%* | N=75  56s (40-85) | N=16  44s (20-159) | N=13  58s (34.5-101.5) | N=12  96s (26-133) | N=18  148s (79-238) | N=16  49s (32-65) |
| Verbal documentation  *N: patient cases with verbal documentation*  *Median duration in seconds, Bootstrap CI 95%* | N=94  12s (9-16) | N=23  14s (10-19.5) | N=12  9.5s (7-26.5) | N=16  11.5s (8-19) | N=20  16s (7-23) | N=23  11s (6-17) |
| Median Interruption-rate (interruptions/hour)  All interruptions  Audio interruptions  Image interruption  Simultaneous speaking  Other interruptions  All Questions:  Image related questions  Patient Information  Organizational matters  Attendance | 78.16  25.24  24.83  8.24  4.6  18.12  1.79  11.47  1.79  1.76 | 97.89  29.99  37.04  12.35  2.65  15.87  1.76  11.46  0.88  1.76 | 99.88  25.24  30.73  6.59  7.68  29.63  2.20  19.76  2.20  5.49 | 78.16  20.23  24.83  3.68  4.60  24.83  0.92  17.47  0.92  5.52 | 51.07  8.79  6.59  8.24  9.33  18.12  3.29  10.98  2.75  1.10 | 73.18  27.66  21.42  10.71  1.78  11.60  1.78  7.14  1.78  0.89 |
